# Sleep disturbances, shift work, and epigenetic ageing in working-age adults: findings from the Young Finns study

**DOI:** 10.1186/s13148-025-01860-w

**Published:** 2025-04-02

**Authors:** Ida Autio, Aino Saarinen, Saara Marttila, Emma Raitoharju, Pashupati P. Mishra, Nina Mononen, Mika Kähönen, Liisa Keltikangas-Järvinen, Olli Raitakari, Terho Lehtimäki

**Affiliations:** 1 Department of Psychology, Faculty of Medicine, University of Helsinki, Haartmaninkatu 3, P.O. Box 21, 00014 Helsinki, Finland; 2 Molecular Epidemiology, Faculty of Medicine and Health Technology, Cardiovascular Research Center Tampere, Tampere University, Tampere, Finland; 3 Gerontology Research Center, Tampere University, Tampere, Finland; 4 Department of Clinical Chemistry, Faculty of Medicine and Health Technology, Cardiovascular Research Center Tampere, Tampere University, Tampere, Finland; 5 Fimlab Laboratories, Department of Clinical Chemistry, Tampere, Finland; 6 Tampere University Hospital, Wellbeing Services County of Pirkanmaa, Tampere, Finland; 7 Department of Clinical Physiology, Faculty of Medicine and Health Technology, Tampere University Hospital, Tampere University, Tampere, Finland; 8 Research Centre of Applied and Preventive Cardiovascular Medicine, University of Turku, Turku, Finland; 9 Centre for Population Health Research, University of Turku, Turku University Hospital, Turku, Finland; 10 Department of Clinical Physiology and Nuclear Medicine, Turku University Hospital, Turku, Finland

**Keywords:** Biological ageing, Epigenetic clock, Shift work, Insomnia, Obstructive sleep apnoea, Chronotype, Sleep quality

## Abstract

**Background:**

Sleep disturbances are known to have adverse effects on health, but knowledge on the effect of sleep disturbances on epigenetic ageing is limited. We investigated (1) whether symptoms of insomnia, obstructive sleep apnoea, sleep deprivation, and circadian rhythm lateness are associated with epigenetic ageing, and (2) whether years spent in shift work moderates these associations.

**Methods:**

We used the population-based Young Finns data (*n* = 1618). Epigenetic clocks such as AgeDev_Hannum_, AgeDev_Horvath_, AgeDev_Pheno_, AgeDev_Grim_, and DunedinPACE were utilized to measure epigenetic ageing. Sleep was evaluated using various validated self-report questionnaires. Covariates included sex, array type, smoking status, health behaviours, socioeconomic factors, and cardiovascular health factors.

**Results:**

Among the various sleep measures, obstructive sleep apnoea symptoms were most consistently linked to accelerated epigenetic ageing, as measured by AgeDev_Grim_ and DunedinPACE. Insomnia, sleep deprivation, and years spent in shift work were not associated with epigenetic ageing after adjusting for health-related or socioeconomic covariates. Additionally, we found interactions between years spent in shift work and sleep disturbances when accounting for epigenetic ageing. Among those with little to no history of shift work, both insomnia and sleep deprivation were associated with more accelerated epigenetic ageing in AgeDev_Grim_ when compared to long-term shift workers. However, the pace of epigenetic ageing (measured with DunedinPACE) appears to be higher in those with both sleep deprivation and longer history of shift work.

**Conclusions:**

Among various sleep measures, symptoms of obstructive sleep apnoea appear to be most consistently associated with accelerated epigenetic ageing even after adjusting for various health-related and socioeconomic factors. Shift work seems to have a crucial role in the relationship between sleep disturbances and epigenetic ageing in working-age adults.

**Supplementary Information:**

The online version contains supplementary material available at 10.1186/s13148-025-01860-w.

## Introduction

Epigenetic ageing measures, also known as epigenetic clocks, are biological indicators of the ageing process. The first-generation clocks—Hannum [1] and Horvath [2] methods—predict chronological age based on CpG methylation patterns in the genome. The second-generation epigenetic clocks, such as PhenoAge [3] and GrimAge [4], were built using a variety of surrogate biomarkers instead of chronological age. The most recent epigenetic ageing measure and the only one built on longitudinal data, DunedinPACE, measures the pace of epigenetic ageing and is based on 19 biomarkers from several organ systems [5]. All epigenetic clocks predict morbidity, mortality, and disability, although the first-generation clocks have limitations in health research due to their more accurate ability to predict chronological age [6–8]. Various health conditions have been linked to accelerated epigenetic ageing, but the relationship between sleep disturbances and epigenetic clocks has received less research attention.

Sleep disturbances, including insomnia, obstructive sleep apnoea, and excessive daytime sleepiness, are known to be common in the general population. It has been reported that up to 40% of the population are dissatisfied with their quantity of sleep, while 16–21% often suffer from difficulty falling asleep or maintaining sleep [9]. The prevalence of diagnosed insomnia has been estimated to be 4.4–11.7% [9], while prevalence estimates of obstructive sleep apnoea vary between 9 and 38% [10]. It is also known that sleep disorders, sleep quality, and sleep quantity are associated with numerous health conditions, including type II diabetes, hypertension, hypercholesterolemia, coronary heart disease, and possibly cognitive decline and dementia [11–17]. These findings, among others, indicate a significant public health burden surrounding sleep disturbances. This study focuses on insomnia, sleep deprivation, symptoms of obstructive sleep apnoea, and chronotype while also considering years in shift work.

Regarding the association between sleep disturbances and epigenetic ageing, evidence is still limited. Insomnia symptoms have previously been found to be associated with accelerated epigenetic ageing as measured by GrimAge and accelerated pace of epigenetic ageing as measured by DunedinPACE [18] as well as accelerated extrinsic epigenetic ageing (EEAA, Hannum method) [19]. Insomnia was also associated with accelerated DunedinPACE in a previous study [20]. Regarding sleep duration and epigenetic ageing, the results have been contradictory: some studies report an association between shorter sleep duration and accelerated epigenetic ageing [18, 21], others have found no association [19, 22], but longer sleep duration has also been linked to accelerated epigenetic ageing [23]. Sleep disordered breathing, of which obstructive sleep apnoea is considered to be a severe form, has been found to be associated with accelerated epigenetic ageing as measured by PhenoAge in a study including only older adults [24]. There is also some evidence that epigenetic clocks can reverse after treatment for obstructive sleep apnoea [25]. Regarding chronotype, an earlier chronotype has been found to be associated with slower epigenetic ageing as measured by GrimAge in older men [26]. Most existing studies on epigenetic ageing and sleep have small sample sizes (*n* < 50), only include women or men, or only include older adults (aged over 50 years).

To date, no study has examined possible interactions between sleep disturbances and shift work on epigenetic ageing. Shift work is known to commonly co-occur with sleep disturbances [27]. Shift work has been found to be associated with all-cause mortality, immunological issues, obesity, metabolic syndrome, cardiovascular disease, depression, cancer mortality, and all-cause mortality [28, 29]. Previous studies also suggest that shift work is associated with accelerated epigenetic ageing, especially among women [30, 31]. There is evidence for interactions between sleep disturbances with shift work when predicting various health outcomes, such as cancer risk [32], chronic kidney disease [33], periodontal disease [34], or morbidity [35]. However, interactions between sleep and shift work have not been investigated in relation to epigenetic ageing.

This study aims to examine the relationship between epigenetic ageing and various sleep disturbances in a population-based sample of working-aged Finnish adults (aged 34–49 years). Second, we aim to examine whether shift work is associated with epigenetic ageing and whether shift work modifies the associations between sleep disturbances and epigenetic ageing. Our sleep measures include insomnia symptoms, sleep deprivation, symptoms of obstructive sleep apnoea and circadian rhythm lateness. In addition, our data enabled us to adjust for numerous possible confounders, including health-related factors such as diagnosed hypertension, blood pressure, the presence of cardiovascular disease such as congestive heart failure and history of stroke, BMI, alcohol use and physical activity as well as socioeconomic factors including education and income.

## Methods

### Participants

The Young Finns Study (YFS) is an ongoing prospective follow-up study that has begun in 1980 (baseline assessment), and follow-ups have been conducted in 1983, 1986, 1989, 1992, 1997, 2001, 2007, 2011/2012, and 2018–2020. Altogether 4320 subjects were invited (born in 1962, 1965, 1968, 1971, 1974, or 1977), and 3596 of them participated in the baseline study. The sampling was designed to include a population-based sample of non-institutionalized Finnish children, representative with regard to most crucial sociodemographic factors. In practice, the sampling was conducted in collaboration of five Finnish universities with medical schools (i.e. Universities of Helsinki, Turku, Tampere, Oulu, and Kuopio). A more detailed description of the YFS can be found elsewhere [36].

Of the 3596 participants, we first excluded 1885 participants who had no data on epigenetic clocks. Later, participants were excluded if they had no data available on the sleep measure of interest or any of the covariates in a particular model. Out of participants who had data available on epigenetic clocks, 93 did not have data on minimal covariates, 250 had no data on all health covariates and 270 had no data on all socioeconomic covariates. The sample size varied between 1439 and 1618 in analyses regarding sleep measures, and between and 581 and 716 in analyses regarding shift work.

### Indicators of epigenetic ageing

Epigenetic ages were calculated for blood samples from 2011. Genome-wide DNA methylation levels from whole blood were obtained with Illumina Infinium HumanMethylation450 BeadChip (*n* = 182) or Illumina Infinium MethylationEPIC BeadChip (*n* = 1529) following standard protocol by Illumina. Preprocessing and normalization of the methylation data have been described in detail elsewhere [37].

Indicators of epigenetic age included in the study were the Horvath clock [2], Hannum clock [1], PhenoAge [3] and GrimAge [4]. For all these clocks, we utilized the measure of epigenetic age deviation, which is defined as the residual that results from regressing epigenetic age on chronological age [38]. These are denoted as AgeDev_Horvath_, AgeDev_Hannum_, AgeDev_Pheno_, and AgeDev_Grim_. We also included a measure for pace of ageing, DunedinPACE [5]. In sensitivity analyses, we used the derivatives of the Horvath and Hannum clocks, IEAA_Horvath_, IEAA_Hannum_, and EEAA_Hannum_ [38] as well as principal component (PC)-based epigenetic clocks including AgeDevPC_Pheno_, AgeDevPC_Grim_, AgeDevPC_Hannum_, and AgeDevPC_Horvath_ [39]. All measures of epigenetic age deviation or pace of epigenetic ageing were calculated according to published methods described above. Pearson correlations between different measures of epigenetic ageing can be found in Supplementary Fig. 1.

### Sleep measures

Participant responses for all sleep measures (except for circadian rhythm lateness) were gathered in both 2007 and 2011. In the final analyses, we used the average scores between the measurement years because, first, also previous studies on sleep and epigenetic ageing have averaged sleep scores if data was available on multiple measurement years [22]. Second, evidence from intervention studies suggests that lifestyle factors need to be examined over periods of years to observe changes in epigenetic clocks [40]. Third, because there were some missing values in 2007 and 2011, using the average scores allowed us to increase the sample size and thereby the statistical power of our analyses.

**Insomnia symptoms** were measured using Jenkins Sleep Scale (JSS) [41] in 2007 and 2011, capturing the frequency and severity of symptoms. JSS includes four items (e.g. “During the past month, how often have you experienced trouble falling asleep?”) with a 6-point Likert scale (1 = “not at all”, 6 = “every night”). JSS has demonstrated high internal consistency and its reliability and construct validity appear to be good [42]. Additionally, the JSS has shown good predictive validity in various health outcomes, including weight gain [43] and type II diabetes [15]. The Finnish translation of the JSS is also found to have adequate internal consistency and construct validity in a previous cohort study [44]. In this study, an averaged score of the JSS items was calculated for 2007 and 2011 separately (if the participant had responded to at least 50% of the items), with higher scores indicating more severe insomnia symptoms. Then, the scores for both years were averaged. If the score for only one year was available, it was used instead. The value of Cronbach’s alpha was *α *= 0.77 in the 2007 survey and *α *= 0.76 in the 2011 survey, indicating high consistency between items in both surveys and thus sufficient reliability. Participants’ scores in 2007 and 2011 showed moderate correlation (Pearson’s *r* = 0.54, *p* < 0.001), indicating moderate stability.

**Symptoms of obstructive sleep apnoea** were measured using Epworth Sleepiness Scale (ESS) [45] and three additional items more specific to obstructive sleep apnoea. ESS measures excessive daytime sleepiness, which can be related to disorders such as obstructive sleep apnoea and narcolepsy. ESS was used in follow-ups of 2007 and 2011, and it includes eight items measuring the tendency to fall asleep in various everyday situations (e.g. “How likely will you fall asleep when you are watching TV?”), and answers are given with a 4-point Likert scale (0 = “I would never doze”, 4 = “a high chance of dozing”). The internal consistency of the ESS has been found to be good [46]. In our sample, the Cronbach’s alpha for ESS was *α* = 0.72 in both 2007 and 2011, indicating high consistency between items. ESS has previously correlated with other measures of sleep apnoea and the measure seems to have good test–retest reliability in non-clinical samples [47]. In addition to the items of the ESS, three other items from 2007 and 2011 were included in the sleep apnoea symptom measure of this study. They were related to frequency of snoring (1 = “once a month or less”, 5 = “every night or nearly every night”), quality of snoring (1 = “I do not snore”, 5 = “loud and uneven snoring”) and frequency of episodes of stopped breathing during sleep (1 = “once a month or less”, 5 = “every night or nearly every night”). Average scores of the eight ESS items were calculated for 2007 and 2011, with higher scores indicating more severe symptoms. The scores of 2007 and 2011 were then averaged. The same process was then repeated for the three additional items. Then, the ESS average and the average score of the three other items were standardized. Finally, the two standardized scores were averaged. Participants were excluded from analyses if responses were unavailable for more than 50% of items in both years. Participants’ sleep apnoea symptoms scores in 2007 and 2011 showed high correlation (Pearson’s *r* = 0.73, *p* < 0.001), indicating high stability.

A **sleep deprivation** score was computed for both 2007 and 2011 as the difference between self-reported optimal amount of sleep and actual amount of sleep. For the latter, participants were instructed to report their usual amount of sleep (reported as 5 h or less, 6 h, 6.5 h… 10 h or more). Scores for both years were then averaged. Existing evidence suggests that both sleep deprivation and excessive sleeping may be associated with poor health outcomes, including diabetes [48], indicating curvilinear associations between sleep deprivation and health outcomes. A curvilinear association has been reported when examining sleep deprivation and phenotypic age [49]. Therefore, the sleep deprivation score was also squared (with high values indicating both sleep deprivation and hypersomnia) and used as a quadratic term in additional models. In addition, we utilized a measure of sleep duration, calculated as the average of actual amount of sleep in 2007 and 2011. The responses between the measurement years showed moderate stability (Pearson’s *r* = 0.43, *p* < 0.001, and Pearson’s *r* = 0.56, *p* < 0.001, for sleep deprivation and sleep duration, respectively).

**Circadian rhythm lateness** was measured with a shortened version of the Morningness-Eveningness Questionnaire (MEQ) [50]. The predictive validity of MEQ is good since it has been found to be associated with various metabolic biomarkers among those with type II diabetes [51] as well as health behaviours and cardiovascular health among women [52]. The measure used in this study includes six selected items from the MEQ (items 4, 5, 9, 15, 17, and 19; e.g. “How easy do you find it to wake up in the morning (when you are not woken up unexpectedly)?”) and responses were given using a 4- or 5-point Likert scale (five items and one item, respectively). Responses were available from 2011, and the value for Cronbach’s alpha was *α* = 0.81, indicating high consistency between items. Each item was first standardized. Then, an average score was calculated for all available items so that higher scores indicate a later circadian rhythm. Participants were excluded from analyses if they had no available data for 50% or more of items. Pearson correlations between different sleep measures can be found in Supplementary Fig. 1.

### Covariates

The data on all covariates were gathered in 2011. All our models were adjusted for sex, self-reported smoking status (daily smoking vs. not) and DNA array type as these have been found to be associated with differences in DNA methylation and the pace of epigenetic age acceleration [6]. In addition, we utilized a set of health-related and socioeconomic covariates as described below.

**A Physical activity index** was based on a questionnaire including questions on the frequency and intensity of physical activity, frequency of vigorous physical activity, time spent on vigorous exercise (in hours), participation in organized physical activity, and average duration of a physical activity session. A more detailed description of the index and its creation can be found elsewhere [53].

To construct **an alcohol consumption index**, participants were asked to report their consumption of different alcohol beverages during the past week. The volumes were then summed to determine consumption measured in alcohol units (1 unit = 14 g of alcohol). The final categorization was done based on daily alcohol consumption (average of the week) as follows: 1: no alcohol consumption during the past week, 2: > 0 to < 2 units per day, 3: 2 to < 4 units per day, and 4: ≥ 4 units per day. The creation of this alcohol consumption index has been described in more detail elsewhere [54].

**Other health covariates** included diagnosed hypertension, systolic blood pressure, diastolic blood pressure, cardiovascular disease status, diabetes, and BMI. Diagnosed hypertension was self-reported by participants (0 = no, 1 = yes). Diastolic and systolic blood pressure were also included as covariates in order to better account for possible undiagnosed cases of hypertension. Blood pressure was measured in sitting position after 5-min rest. A mercury sphygmomanometer at phases 1 and 2 and with a random zero sphygmomanometer (Hawksley & Sons Ltd) at phase 3 was used. Cuff size for the measurement covered two-thirds of the participant’s arm length. Korotkoff’s first phase was determined as the indicator of systolic blood pressure. Readings to the nearest even number of millimetres of mercury were conducted 3 times for each participant. In the analyses, the average values of diastolic and systolic blood pressure were used between the three measurements. Cardiovascular disease status was considered positive (= 1) if the participant self-reported a history of stroke, chest pain related to coronary heart disease, congestive heart failure, coronary artery bypass surgery, or coronary angioplasty (all reported as 0 = no, 1 = yes). Otherwise, cardiovascular disease status was coded as 0. Diabetes was also self-reported and included in models separately (0 = no, 1 = yes). Separate items for type I and type II diabetes were combined: if either of these was reported as 1, the diabetes variable was coded as positive.

**Socioeconomic covariates** included gross yearly income, years of education and working hours (regular daytime job vs. not), all of which were self-reported. Gross yearly income was reported with a 13-point Likert scale (1 =  < 5 000 €, 13 =  > 60 000€). The education variable indicates years of education, including years of vocational training. Working hours during the past 12 months were reported with six categories (regular daytime job, shift work with two rotating shifts, shift work with three rotating shifts, fixed evening or nighttime working hours, irregular working hours, and not working outside home). The variable of working hours was dichotomized: regular daytime job (= 0) or shift work (= 1), which included all other categories except those not working outside home (coded as missing). Thus, those not working outside home were excluded from models where working hours were included as a covariate. In analyses regarding shift work, a measure of years in shift work was used. Participants freely self-reported the total amount of years they have spent in shift work. This resulted in a range of 0–30 years.

### Statistical models

All analyses were conducted using R (versions 4.3.1 and 4.4.0). Stata MP 18.0 was used for plotting.

The associations between the sleep measures and epigenetic ageing measures were examined with linear regression models. Separate models were estimated for each epigenetic ageing measure (AgeDev_Hannum_, AgeDev_Horvath_, AgeDev_Pheno_, AgeDev_Grim_, and DunedinPACE). Each sleep measure was added as predictor separately (insomnia symptoms, sleep apnoea symptoms, sleep deprivation, and circadian rhythm lateness). There is evidence that health-related and socioeconomic factors seem to mediate the association between sleep and health [55]. Accordingly, to gain insight into possible mediating mechanisms between sleep disturbances and epigenetic ageing, we ran the regression analyses using three models with partially different sets of covariates (Models 1, 2, and 3). Model 1 only included minimal covariates (sex, daily smoking status, and DNA array type). Model 2 was additionally adjusted for health variables (BMI, cardiovascular disease status, diabetes, hypertension, systolic and diastolic blood pressure, alcohol use, and physical activity). Model 3 was adjusted for minimal covariates and socioeconomic covariates (gross yearly income, years of education, and regular daytime job vs. shift work).

To account for multiple testing, we used the false discovery rate (FDR) correction with the Benjamini–Hochberg method to adjust *p*-values [56]. The analyses were conducted for the whole sample, as no statistically significant sex interactions were observed while examining the associations between sleep measures and epigenetic ageing (*p* > 0.05).

Next, we examined whether years in shift work modify the associations between sleep measures and epigenetic ageing. Separate models were again estimated for each sleep measure. An interaction term between each sleep measure and years in shift work was added to the models, utilizing the same sets of covariates (Models 1, 2 and 3).

In order to assess the robustness of the results, the analyses were repeated using only cases where Illumina Infinium MethylationEPIC BeadChip was used for DNA methylation profiling. Additionally, the main analyses were repeated with IEAA_Hannum_, IEAA_Horvath_, and EEAA_Hannum_ and four principal component (PC)-based clocks designed for reduction in technical noise and increased reliability [39]. These are denoted as AgeDevPC_Pheno_, AgeDevPC_Grim_, AgeDevPC_Hannum_, and AgeDevPC_Horvath_.

Attrition over the follow-up period was examined in order to evaluate possible differences between included and dropped-out participants. This was done using independent samples t-tests (for continuous variables) and chi-square tests (for categorical variables).

## Results

### Descriptive statistics

Descriptive statistics on demographic variables, sleep measures, epigenetic ageing measures, and covariates are shown in Table 1. Our attrition analyses showed that compared to included participants, dropped-out participants had a lower proportion of women (41.9% vs. 56.1%), a higher proportion of daily smokers (19.4% vs. 14.3%), and slightly higher averaged scores on Jenkins Sleep Scale (2.34 vs. 2.26). There were no statistically significant differences in other sleep measures or health variables. Additional details can be found in Supplementary Table 1.

**Table 1:** Descriptive characteristics of the included population

	Proportion (%)	Mean (SD)	Range (min, max)
Age (2011)		42.04 (4.98)	34.00, 49.00
Sex (Female)	56.1		
*Health variables*			
Daily smoking status	14.3		
Physical activity index		9.05 (1.88)	5.00, 15.00
Alcohol consumption		0.80 (1.19)	0.00, 14.29
BMI		26.59 (4.99)	17.47, 58.47
Hypertension, diagnosis	9.1		
Systolic blood pressure		118.80 (13.91)	83.33, 178.67
Diastolic blood pressure		74.76 (10.47)	44.00, 113.33
Cardiovascular disease status	0.7		
Diabetes	2.6		
*Socioeconomic variables*			
Years of education		15.32 (3.56)	8.00, 30.00
Gross annual income		7.37 (3.08)	1.00, 13.00
Irregular working hours	31.1		
*Sleep measures*			
Jenkins Sleep Scale (JSS)*		9.00 (3.62)	4.00, 24.00
Epworth Sleepiness Scale (ESS)*		5.43 (3.20)	0.00, 20.00
Sleep deprivation score		0.49 (0.74)	− 2.00, 4.00
Morningness-Eveningness Questionnaire (MEQ; shortened)*		12.98 (3.40)	6.00, 24.00
*Measures of epigenetic ageing/pace of ageing*			
AgeDev_Pheno_		0.11 (5.35)	− 17.47, 20.11
AgeDev_Grim_		− 0.03 (3.70)	− 9.21, 16.14
AgeDev_Hannum_		0.07 (4.17)	− 19.22, 14.17
AgeDev_Horvath_		0.07 (4.18)	− 22.69, 19.49
DunedinPACE		0.94 (0.10)	0.61, 1.35

All participants included in at least one model were included in this table (*n* = 1618)

^*^Sum variables (averaged from 2007 and 2011) reported, but variables used in analyses were averaged across items (JSS) or standardized item by item and averaged (ESS, MEQ). MEQ was coded so that larger values indicate later chronotypes

### Main analyses

First, we examined the main effect of insomnia symptoms on epigenetic ageing measures. The results are shown in Table 2. An association between insomnia and epigenetic age acceleration as measured by DunedinPACE was observed in Model 1 (*β* = 0.01, *p* = 0.007), but not in Model 2 (with added health-related covariates) or Model 3 (with added socioeconomic covariates). No associations were observed between insomnia and the other epigenetic clocks, including AgeDev_Pheno_, AgeDev_Grim,_ AgeDev_Hannum_, and AgeDev_Horvath_ (Table 2) in Models 1, 2, or 3. The results of analyses regarding Horvath and Hannum clock derivatives are displayed in Supplementary Table 2.

**Table 2:** Results of regression analyses when predicting epigenetic ageing measures by insomnia symptoms

		Model 1 (*n* = 1617)			Model 2 (*n* = 1451)			Model 3 (*n* = 1440)	
	*β*	95% CI	*p*	*β*	95% CI	*p*	*β*	95% CI	*p*
AgeDev_Pheno_	0.15	− 0.14, 0.44	0.307	0.02	− 0.28, 0.33	0.888	0.14	− 0.17, 0.45	0.379
AgeDev_Grim_	0.07	− 0.09, 0.23	0.365	− 0.07	− 0.24, 0.09	0.380	0.00	− 0.16, 0.17	0.959
AgeDev_Hannum_	− 0.05	− 0.28, 0.17	0.635	− 0.13	− 0.37, 0.11	0.299	− 0.07	− 0.31, 0.17	0.559
AgeDev_Horvath_	0.05	− 0.17, 0.28	0.650	− 0.07	− 0.31, 0.18	0.594	− 0.02	− 0.26, 0.22	0.867
DunedinPACE	0.01	0.00, 0.01	0.007*	0.00	0.00, 0.01	0.199	0.00	0.00, 0.01	0.112

Statistically significant associations (*p* < 0.05, unadjusted) are bolded, and those that remained significant after FDR correction are marked with an asterisk. Model 1 was adjusted for sex, daily smoking status, and array type. Model 2 was adjusted for Model 1 covariates and health factors. Model 3 was adjusted with Model 1 covariates and socioeconomic factors

Next, we examined the association of sleep deprivation with epigenetic ageing. In Models 1, sleep deprivation was associated with AgeDev_Horvath_ (*β* = 0.45, *p* = 0.028) (Table 3). The sleep deprivation variable was then added to the models as an additional quadratic term, as previous studies suggest that both excessive sleeping and lack of sleep are associated with adverse health outcomes [48]. The quadratic sleep deprivation term was associated with AgeDev_Grim_ Model 1 (*β* = 0.17, *p* = 0.029), but not after correction for multiple testing (*p* = 0.058). The quadratic sleep deprivation term was not associated with any other epigenetic ageing measure in Models 1, 2, or 3 (Supplementary Table 3).

**Table 3:** Results of regression analyses when predicting epigenetic ageing measures by sleep deprivation

		Model 1 (*n* = 1616)			Model 2 (*n* = 1451)			Model 3 (*n* = 1439)	
	*β*	95% CI	*p*	*β*	95% CI	*p*	*β*	95% CI	*p*
AgeDev_Pheno_	0.14	− 0.38, 0.66	0.588	0.09	− 0.44, 0.63	0.734	0.24	− 0.30, 0.78	0.384
AgeDev_Grim_	− 0.15	− 0.44, 0.13	0.287	− 0.14	− 0.43, 0.15	0.331	− 0.07	− 0.36, 0.22	0.638
AgeDev_Hannum_	0.02	− 0.38, 0.42	0.923	− 0.05	− 0.47, 0.38	0.832	0.04	− 0.39, 0.46	0.867
AgeDev_Horvath_	0.45	0.05, 0.85	0.028*	0.37	− 0.06, 0.80	0.090	0.39	− 0.04, 0.81	0.074
DunedinPACE	0.00	− 0.01, 0.01	0.552	0.01	0.00, 0.02	0.082	0.01	0.00, 0.02	0.206

Statistically significant associations (*p* < 0.05, unadjusted) are bolded, and those that remained significant after FDR correction are marked with an asterisk. Model 1 was adjusted for sex, daily smoking status, and array type. Model 2 was adjusted for Model 1 covariates and health factors. Model 3 was adjusted with Model 1 covariates and socioeconomic factors

In addition to sleep deprivation, we examined the association between sleep duration (hours of sleep) and measures of epigenetic ageing. The results are displayed in Supplementary Table 7. To summarize, longer sleep duration was associated with lower DunedinPACE in Model 1 (*β* = –0.01, *p* < 0.001), Model 2 (*β* = –0.01, *p* = 0.001), and Model 3 (*β* = –0.01, *p* < 0.001). We also found an association between hours of sleep and AgeDev_Grim_ in Model 1, but this association did not sustain after FDR correction.

For circadian rhythm lateness, no effects were observed for any epigenetic ageing measure (*p* = 0.083–0.769). The results of these analyses can be found in more detail in Supplementary Table 4.

**Fig. 1:**
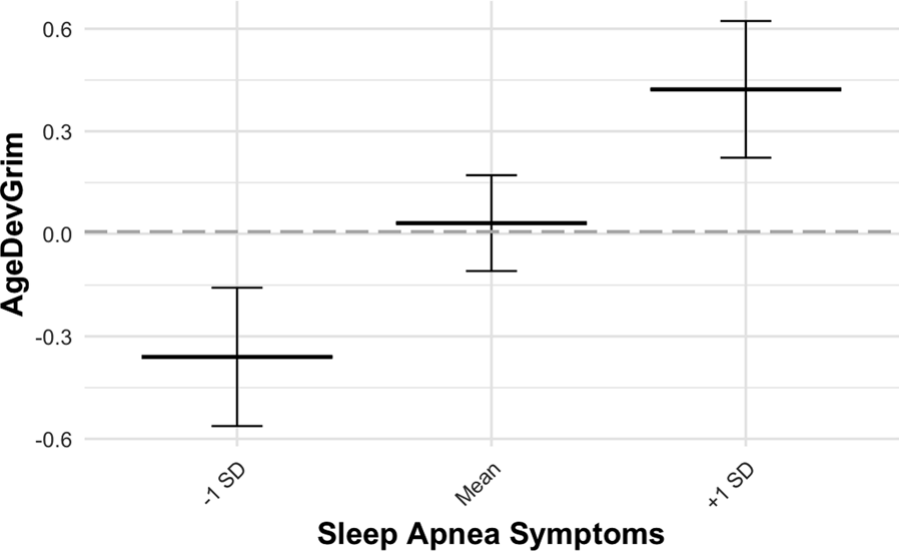
Estimated differences in AgeDev_Grim_ by the level of sleep apnoea symptoms. Differences were estimated separately for participants with low (− 1 SD) mean or high (+ 1 SD) levels of sleep apnoea symptoms. The model was adjusted for sex, array type, and smoking status

Symptoms of obstructive sleep apnoea were found to be associated with epigenetic age deviation in AgeDev_Grim_ in Model 1 (*β* = 0.51, *p* < 0.001), Model 2 (*β* = 0.24, *p* = 0.036), and Model 3 (*β* = 0.44, *p* < 0.001). Figure 1 displays differences in AgeDev_Grim_ when participants were grouped based on their sleep apnoea symptom score. Similarly, sleep apnoea symptoms were associated with pace of epigenetic ageing as measured by DunedinPACE in Model 1 (*β* = 0.02, *p* < 0.001), Model 2 (*β* = 0.01, *p* = 0.030), and Model 3 (*β* = 0.02, *p* < 0.001). In Models 1, sleep apnoea symptoms were also associated with AgeDev_Pheno_ (*β* = 0.42, *p* = 0.030), AgeDev_Horvath_ (**β** = 0.29, *p* = 0.046), and IEAA_Horvath_ (*β* = 0.29, *p* = 0.045). The results are displayed in more detail in Table 4.

**Table 4:** Results of regression analyses when predicting epigenetic ageing measures by sleep apnoea symptoms

		Model 1 (n = 1618)			Model 2 (n = 1452)			Model 3 (n = 1441)	
	*β*	95% CI	*p*	*β*	95% CI	*p*	*β*	95% CI	*p*
AgeDev_Pheno_	0.42	0.07, 0.77	0.018*	0.13	− 0.25, 0.51	0.488	0.39	0.03, 0.76	0.036*
AgeDev_Grim_	0.51	0.32, 0.70	1.21e-7*	0.24	0.04, 0.44	0.019*	0.44	0.25, 0.64	1.10e-5*
AgeDev_Hannum_	− 0.06	− 0.33, 0.21	0.646	− 0.21	− 0.51, 0.09	0.171	− 0.13	− 0.41, 0.16	0.386
AgeDev_Horvath_	0.29	0.02, 0.56	0.037*	0.18	− 0.12, 0.49	0.236	0.16	− 0.13, 0.45	0.269
DunedinPACE	0.02	0.02, 0.03	1.32e-12*	0.01	0.00, 0.02	0.014*	0.02	0.02, 0.03	3.27e-10*

Statistically significant associations (*p* < 0.05, unadjusted) are bolded, and those that remained significant after FDR correction are marked with an asterisk. Model 1 was adjusted for sex, daily smoking status, and array type. Model 2 was adjusted for Model 1 covariates and health factors. Model 3 was adjusted with Model 1 covariates and socioeconomic factors

### Sensitivity analyses

The analyses were first repeated so that only cases with Illumina Infinium MethylationEPIC BeadChip were included (*n* = 1529). All other results were replicated, except for the associations between sleep deprivation and AgeDev_Horvath_ (please see Supplementary Table 5).

We also reran the analyses using principal component (PC) clocks. Again, we found an association between sleep apnoea symptoms and AgeDevPC_Grim_ (*p* = 4.44e-7). The association between sleep apnoea symptoms and AgeDev_Pheno_, however, was not replicated when using AgeDevPC_Pheno_ (*p* = 0.251). Finally, we reran the analyses using the Hannum and Horvath clock derivatives IEAA_Horvath_, IEAA_Hannum_, and EEAA_Hannum_. The main results were replicated: We found associations between sleep deprivation and IEAA_Horvath_ (*β* = 0.48, *p* = 0.018) and between sleep apnoea symptoms and IEAA_Horvath_ (*β* = 0.29, *p* = 0.033). The results of these analyses can be found in more detail in Supplementary Table 2.

### The moderating effect of shift work on the associations of sleep and epigenetic ageing

First, we examined whether years in shift work are associated with epigenetic ageing. Years of shift work had a main effect on DunedinPACE in Model 1 (*β* = 0.001, *p* = 0.016). A similar effect in Model 2 did not remain statistically significant after multiple testing correction (*p* = 0.077). The results are displayed in more detail in Supplementary Table 6.

**Fig. 2:**
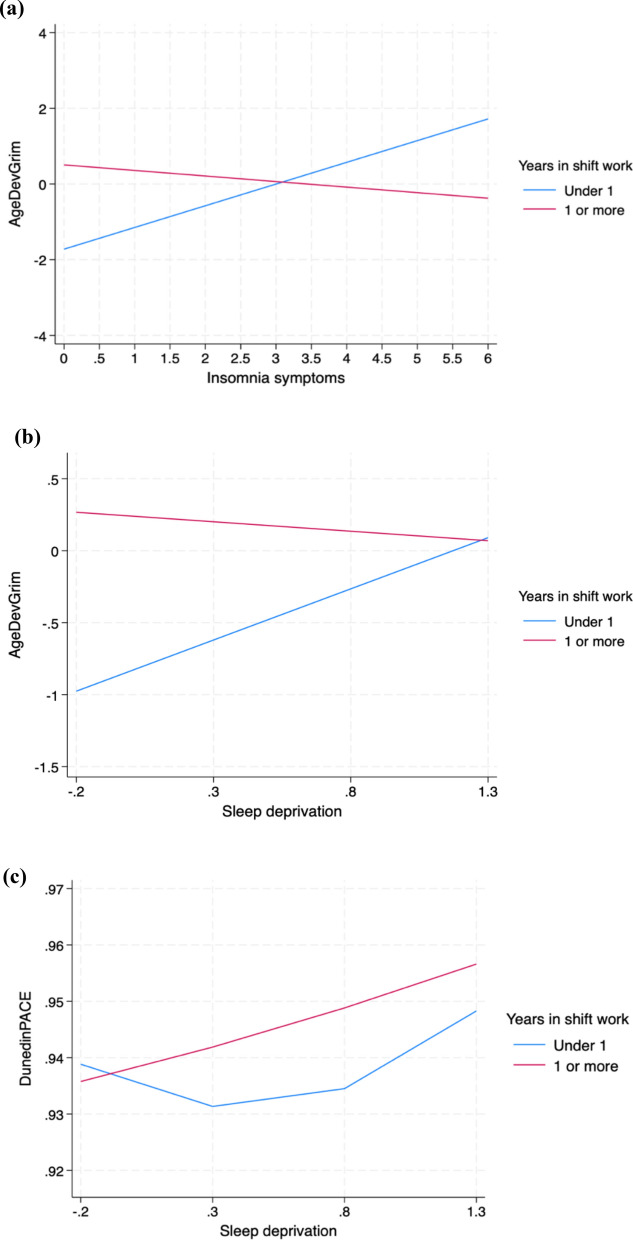
Interaction effects between years of shift work and sleep measures. Interaction effects between years of shift work and insomnia symptoms (**a**), sleep deprivation (**b**), and the quadratic term of sleep deprivation (**c**) when predicting AgeDev_Grim_ or DunedinPACE. (**c**) was adjusted for array type, sex, and smoking status. (**a**) and (**b**) were additionally adjusted for health covariates

Finally, we examined whether years spent in shift work could moderate the associations between the sleep measures and epigenetic ageing. Thus, we examined interaction effects between each sleep measure and years spent in shift work when predicting indicators of epigenetic ageing. An interaction between insomnia and years in shift work was observed when predicting AgeDev_Grim_ in Model 2 (*β* = − 0.04, *p* = 0.012) (see Fig. 2a). When predicting AgeDev_Grim_ with sleep deprivation, there was an interaction effect between sleep deprivation and years in shift work in Model 2 (*β* = − 0.05, *p* = 0.006) (see Fig. 2b). Similarly, when predicting DunedinPACE with the quadratic sleep deprivation model, there was an interaction effect with years in shift work in Model 1 (*β* = − 0.001, *p* = 0.028) (see Fig. 2c). No other significant interaction effects were found between years in shift work and sleep measures when predicting epigenetic ageing.

Taken together, the interaction effects indicated that the associations of insomnia and sleep deprivation with accelerated epigenetic ageing as measured by AgeDev_Grim_ seem to be stronger in those with little to no history of shift work (Fig. 2a and b). When the quadratic sleep deprivation term was included, sleep deprivation seemed to be associated with a more accelerated pace of epigenetic ageing as measured with DunedinPACE in those with over one year of shift work history (Fig. 2c).

## Discussion

This study is the first to examine the associations between multiple sleep measures, years in shift work, and epigenetic ageing in a population-based sample of working-age adults. Insomnia, symptoms of obstructive sleep apnoea, and sleep deprivation were associated with accelerated epigenetic ageing. When considering health-related and socioeconomic covariates, the effect of obstructive sleep apnoea symptoms seemed to be the most robust. The sleep measures predicted epigenetic ageing most consistently in AgeDev_Pheno_, AgeDev_Grim_, and DunedinPACE. In addition, years spent in shift work were associated with accelerated pace of epigenetic ageing. Insomnia and sleep deprivation had different effects on epigenetic ageing depending on years in shift work.

Among various sleep measures, symptoms of obstructive sleep apnoea had the most consistent associations with epigenetic ageing. Also, the associations between obstructive sleep apnoea and AgeDev_Grim_ and DunedinPACE sustained after controlling for a range of health-related, and socioeconomic factors not previously examined, including cardiovascular disease status, diagnosed hypertension and blood pressure. Therefore, symptoms of obstructive sleep apnoea appear to be associated with accelerated epigenetic ageing independently from health-related factors such as hypertension, cardiovascular disease and socioeconomic factors. Previously, sleep apnoea has been found to be associated with alterations in cellular immunity [57–59], but it has also been reported that there are no differences in methylation-inferred blood cell composition between patients with obstructive sleep apnoea and healthy controls [25]. The compositions are also reported not to change longitudinally after sleep apnoea treatment. This indicates that our finding is not explained by differences in peripheral blood counts.

Insomnia has previously been reported to be associated with accelerated DunedinPACE and GrimAge [18, 20]. Since we only observed an effect in DunedinPACE when using minimal covariates (DNA chip type, smoking status, and sex), the association may be partially explained by other health-related or socioeconomic variables not previously considered, including physical activity and hypertension. It is also possible that age could influence the relationship between insomnia and epigenetic ageing, since earlier studies have mostly included older adults.

Insomnia had a stronger accelerating effect on AgeDev_Grim_ in those with little to no history of shift work (when compared to longer-term shift workers). This suggests the possibility of an adaptation effect to shift work over long periods of time. This is in accordance with a previous study reporting accelerated epigenetic ageing among short-term night shift workers (2–6 years) but not among long-term night shift workers [31]. Additionally, insomnia among shift workers is often attributed to an external factor, while insomnia among non-shift workers may imply broader challenges in managing health and lifestyle, which in turn may possibly further accelerate epigenetic ageing. However, when using DunedinPACE, we had quite an opposite finding: sleep deprivation was associated with more accelerated pace of epigenetic ageing in DunedinPACE in long-term shift workers (when compared to those with at most a one-year history of shift work). Taken together, the results suggest that DunedinPACE and GrimAge may capture the ageing effects of sleep deprivation differently. This is further supported by our finding that years in shift work had a main effect on DunedinPACE but not on any other measure of epigenetic ageing. DunedinPACE has been trained using a measure of pace of ageing over 20 years, capturing a total 19 indicators of organ-system ageing, including BMI, triglycerides, total cholesterol, and waist-hip ratio [5]. It may therefore be able to detect effects of long-term shift work better than GrimAge, which is based on cross-sectional data of smoking pack-years and a set of surrogate blood biomarkers, including adrenomedullin and CRP among others.

Reliance on self-reported data on all of our sleep measures can be considered as a limitation of this study, as objective measures such as polysomnography may describe some aspects of sleep more accurately [48]. This may not be the case for sleep quality, which insomnia symptoms also affect. According to a recent meta-analysis, there is discrepancy in objective and subjective sleep measures especially regarding sleep quality, possibly indicating that objective sleep measures do not capture sleep quality adequately [60]. Self-rated sleep quality and quantity have also been found to be associated with various aspects of cardiovascular health [17, 61] , indicating that self-reported sleep measures have predictive validity when examining health outcomes. Another limitation of our study is related to attrition. Our analyses revealed that included and dropped-out populations had differences in proportion of sexes (included population had a larger proportion of females), smoking status (with dropped-out population including more smokers), and insomnia symptoms (dropped-out population had higher scores). However, especially in insomnia symptoms, the differences were minor by effect size. Dropped-out and included populations were also very similar with regard to cardiovascular health, socioeconomic factors, and other sleep measures.

Third, our study design did not allow for direct examination of causal relationships between sleep measures and measures of epigenetic ageing. However, there is evidence that epigenetic clocks have reversed as a response to treatment of sleep apnoea [25], indicating that this kind of a relationship is the most likely explanation for the effects we observed. In addition, a possible cell-level mechanism has been suggested: sleep disturbances can lead to metabolic and endocrine disturbances, eventually leading to DNA damage which in turn can contribute to biological ageing [62]. Epigenome-wide association studies (EWAS) with a larger sample size could be utilized in the future to clarify the mechanisms behind the association between sleep disturbances and epigenetic ageing. A previous EWAS found numerous methylation sites to be associated with nighttime shift work and overall shift work [30]. These sites were all distinct from the ones used to calculate Hannum and Horvath clocks and PhenoAge.

This study also has notable strengths. Most previous evidence on epigenetic ageing and sleep comes from smaller and more limited samples, but our study allows generalization to the working population. We were also able to consider several sleep measures, measures of epigenetic ageing, and possible confounders including various health conditions and socioeconomic factors. In addition, the modifying effect of years spent in shift work has not previously been examined in the context of sleep and epigenetic ageing.

## Conclusion

Insomnia, sleep deprivation, and symptoms of obstructive sleep apnoea predict accelerated epigenetic ageing in the working-age population. Notably, symptoms of obstructive sleep apnoea appear to be associated with accelerated epigenetic ageing independently of a variety of other health-related and socioeconomic factors. Furthermore, our results showed that insomnia and sleep deprivation are related to epigenetic ageing differently among shift workers and non-shift workers. These findings provide new insights to the public health burden posed by sleep disorders, underscoring the importance of addressing obstructive apnoea to mitigate its potential long-term effects on biological ageing.

## Supplementary Information

Additional file1 (DOCX 186 KB)

## Data Availability

The Cardiovascular Risk in Young Finns (YFS) dataset comprises health-related participant data, and their use is therefore restricted under the regulations on professional secrecy (Act on the Openness of Government Activities, 612/1999) and on sensitive personal data (Personal Data Act, 523/1999, implementing the EU data protection directive 95/46/EC). Due to these legal restrictions, the data from this study cannot be stored in public repositories or otherwise made publicly available. However, data access may be permitted on a case by case basis upon request. Data sharing outside the group is done in collaboration with YFS group and requires a data-sharing agreement. Investigators can submit an expression of interest to the chairman of the publication committee (Prof. Mika Kähönen, Tampere University, Finland, mika.kahonen@tuni.fi).
