# Supplementary material for: Sleep disturbances, shift work, and epigenetic ageing in working-age adults: findings from the Young Finns study
Source: Clin Epigenetics. 2025 Apr 2;17:55. doi: 10.1186/s13148-025-01860-w (PMC11966881; doi:10.1186/s13148-025-01860-w)
Supplement: Supplementary file 1 — Additional file1 (DOCX 186 KB) [file 13148_2025_1860_MOESM1_ESM.docx]

**Supplementary Table 1.**

*Differences in characteristics of included and dropped-out participants.*

|  | **Mean difference**  **(dropped-out vs. included)** | **Test statistic** | ***p*** |
| --- | --- | --- | --- |
| Age | 41.26 vs. 42.04 | *t = –*1.47 | 0.142 |
| Sex (Female) | 41.9 % vs. 56.1 % | *χ²* = 6.53 | **0.011*** |
| Daily smoking status | 19.4 % vs. 14.3 % | *χ² =* 5.76 | **0.016*** |
| Physical activity | 8.85 vs. 9.05 | *t* = –1.86 | 0.062 |
| Alcohol consumption | 0.89 vs. 0.80 | *t* = 1.32 | 0.186 |
| BMI | 26.33 vs. 26.59 | *t* = –0.95 | 0.344 |
| Cardiovascular disease status | 0.8 % vs. 0.7 % | *χ²* < 0.01 | 1.000 |
| Hypertension, diagnosis | 9.0 % vs. 9.1 % | *χ²* < 0.01 | 1.000 |
| Systolic blood pressure | 119.48 vs. 118.80 | *t* = 0.89 | 0.372 |
| Diastolic blood pressure | 75.26 vs. 74.76 | *t* = 0.89 | 0.376 |
| Diabetes | 1.9 % vs. 2.6 % | *χ²* = 0.45 | 0.504 |
| Gross annual annual income | 7.38 vs. 7.37 | *t* = 0.08 | 0.934 |
| Years of education | 15.47 vs. 15.32 | *t* = 0.715 | 0.475 |
| Irregular working hours | 34.2 % vs. 31.1 % | *χ²* = 1.13 | 0.288 |
| Jenkins Sleep Scale | 2.34 vs. 2.26 | *t* = 2.02 | **0.043*** |
| Epworth Sleepines Scale | 1.00 vs. 1.02 | *t* = –0.63 | 0.529 |
| Sleep deprivation score | 1.08 vs. 0.95 | *t* = 1.42 | 0.155 |
| Circadian rhythm lateness | 2.20 vs. 2.16 | *t* = 1.21 | 0.224 |

**Supplementary Table 2.**

*Sensitivity analyses using principal component (PC) based epigenetic clocks and Hannum and Horvath clock derivatives.*

|  |  |  | **Insomnia** | | |  | |  | |  | **Sleep deprivation** | | |  | |  | |  | **Circadian rhythm lateness** | | |  | |  | |  | **Sleep apnea symptoms** | | |  | |  | |
| --- | --- | --- | --- | --- | --- | --- | --- | --- | --- | --- | --- | --- | --- | --- | --- | --- | --- | --- | --- | --- | --- | --- | --- | --- | --- | --- | --- | --- | --- | --- | --- | --- | --- |
|  |  | ***β*** | | **(95 % CI)** | ***p*** | |  | | ***β*** | | | **(95 % CI)** | ***p*** | |  | | ***β*** | | | **(95 % CI)** | ***p*** | |  | | ***β*** | | | **(95 % CI)** | ***p*** | |  | |  |
|  |  |  | |  |  | |  | |  | | |  |  | |  | |  | | |  |  | |  | |  | | |  |  | |  | |  |
| AgeDevPC_Pheno_ |  | -0.05 | | -0.29, 0.18 | 0.655 | |  | | 0.14 | | | -0.14, 0.43 | 0.332 | |  | | -0.22 | | | -0.53, 0.08 | 0.144 | |  | | 0.25 | | | 0.00, 0.51 | 0.053 | |  | |  |
| AgeDevPC_Grim_ |  | 0.66 | | -0.01, 0.25 | 0.079 | |  | | 0.12 | | | -0.04, 0.28 | 0.136 | |  | | -0.06 | | | -0.23, 0.11 | 0.491 | |  | | 0.36 | | | 0.22, 0.50 | **4.44e-7*** | |  | |  |
| AgeDevPC_Hannum_ |  | -0.03 | | -0.21, 0.13 | 0.654 | |  | | 0.10 | | | -0.11, 0.31 | 0.354 | |  | | -0.12 | | | -0.34, 0.10 | 0.278 | |  | | 0.05 | | | -0.13, 0.24 | 0.577 | |  | |  |
| AgeDevPC_Horvath_ |  | -0.06 | | -0.24, 0.12 | 0.514 | |  | | -0.11 | | | -0.10, 0.33 | 0.296 | |  | | -0.06 | | | -0.29, 0.17 | 0.620 | |  | | 0.09 | | | -0.11, 0.28 | 0.382 | |  | |  |
| IEAA_Hannum_ |  | -0.01 | | -0.21, 0.19 | 0.933 | |  | | 0.05 | | | -0.32, 0.41 | 0.807 | |  | | -0.04 | | | -0.23, 0.15 | 0.679 | |  | | -0.09 | | | -0.34,0.15 | 0.452 | |  | |  |
| IEAA_Horvath_ |  | 0.08 | | -0.14, 0.30 | 0.490 | |  | | 0.48 | | | 0.08, 0.87 | **0.018*** | |  | | -0.15 | | | -0.36, 0.06 | 0.165 | |  | | 0.29 | | | 0.02,0.55 | **0.033*** | |  | |  |
| EEAA_Hannum_ |  | -0.10 | | -0.37, 0.18 | 0.490 | |  | | -0.02 | | | -0.51, 0.47 | 0.943 | |  | | -0.01 | | | -0.27, 0.25 | 0.914 | |  | | -0.06 | | | -0.39,0.27 | 0.728 | |  | |  |
| PC based epigenetic clocks, the Hannum clock derivatives (IEAA_Hannum_, EEAA_Hannum_), and the Horvath clock derivative (IEAA_Horvath_) were predicted in the regression models using sleep measures. The models were adjusted for array type, sex and smoking status. | | | | | | | | | | | | | | | | | | | | | | | | | | | | | | |  | |  |

**Supplementary Table 3.**

*Results of regression analyses when predicting epigenetic ageing measures by the quadratic sleep deprivation term.*

|  |  |  | **Model 1**  **(n = 1616)** |  |  |  | **Model 2**  **(n = 1451)** |  |  |  | **Model 3**  **(n = 1439)** |  |  |
| --- | --- | --- | --- | --- | --- | --- | --- | --- | --- | --- | --- | --- | --- |
|  |  | ***β*** | **95 % CI** | ***p*** |  | ***β*** | **95 % CI** | ***p*** |  | ***β*** | **95 % CI** | ***p*** |  |
| AgeDev_Pheno_ |  | -0.03 | -0.31, 0.24 | 0.804 |  | -0.03 | -0.33, 0.27 | 0.834 |  | -0.04 | -0.34, 0.26 | 0.782 |  |
| AgeDev_Grim_ |  | 0.17 | 0.02, 0.32 | **0.029*** |  | 0.15 | -0.01, 0.31 | 0.069 |  | 0.11 | -0.06, 0.27 | 0.202 |  |
| AgeDev_Hannum_ |  | -0.04 | -0.26, 0.17 | 0.683 |  | -0.01 | -0.25, 0.23 | 0.942 |  | -0.10 | -0.33, 0.14 | 0.429 |  |
| AgeDev_Horvath_ |  | -0.17 | -0.39, 0.04 | 0.108 |  | -0.15 | -0.39, 0.09 | 0.220 |  | -0.17 | -0.41, 0.07 | 0.158 |  |
| DunedinPACE |  | 0.00 | 0.00, 0.01 | 0.151 |  | 0.00 | -0.01, 0.00 | 0.847 |  | 0.00 | 0.00, 0.01 | 0.487 |  |
| Statistically significant associations (p < 0.05, unadjusted) are bolded, and those that remained significant after FDR correction are marked with an asterisk. Models 1 were adjusted for sex, daily smoking status and array type. Models 2 were adjusted for Model 1 covariates and health factors. Models 3 were adjusted with Model 1 covariates and socioeconomic factors. | | | | | | | | | | | | |  |

**Supplementary Table 4.**

*Results of regression analyses when predicting epigenetic ageing measures by circadian rhythm lateness. The p-values displayed are unadjusted.*

|  |  |  | **Model 1**  **(n = 1617)** |  |  |  | **Model 2**  **(n = 1451)** |  |  |  | **Model 3**  **(n = 1440)** |  |  |
| --- | --- | --- | --- | --- | --- | --- | --- | --- | --- | --- | --- | --- | --- |
|  |  | ***β*** | **95 % CI** | ***p*** |  | ***β*** | **95 % CI** | ***p*** |  | ***β*** | **95 % CI** | ***p*** |  |
|  |  |  |  |  |  |  |  |  |  |  |  |  |  |
| AgeDev_Pheno_ |  | 0.25 | -0.62, 0.12 | 0.181 |  | -0.27 | -0.66, 0.11 | 0.161 |  | -0.28 | -0.67, 0.12 | 0.172 |  |
| AgeDev_Grim_ |  | -0.12 | -0.33, 0.08 | 0.232 |  | -0.10 | -0.30, 0.11 | 0.363 |  | -0.08 | -0.29, 0.14 | 0.474 |  |
| AgeDev_Hannum_ |  | -0.11 | -0.40, 0.18 | 0.446 |  | -0.15 | -0.46, 0.15 | 0.321 |  | -0.14 | -0.45, 0.17 | 0.390 |  |
| AgeDev_Horvath_ |  | 0.09 | -0.20, 0.38 | 0.550 |  | 0.05 | -0.25, 0.36 | 0.741 |  | 0.16 | -0.15, 0.47 | 0.304 |  |
| DunedinPACE |  | -0.01 | -0.01, 0.00 | 0.149 |  | 0.00 | -0.01, 0.00 | 0.313 |  | 0.00 | -0.01, 0.00 | 0.276 |  |
| Statistically significant associations (p < 0.05, unadjusted) are bolded, and those that remained significant after FDR correction are marked with an asterisk. Models 1 were adjusted for sex, daily smoking status and array type. Models 2 were adjusted for Model 1 covariates and health factors. Models 3 were adjusted with Model 1 covariates and socioeconomic factors. | | | | | | | | | | | | |  |

**Supplementary Table 5.**

*Sensitivity analyses with only participants with the Illumina Infinium MethylationEPIC BeadChip (n = 1529) included.*

|  |  |  | **Insomnia** | | |  |  |  | **Sleep deprivation** | | |  |  |  | **Circadian rhythm lateness** | | |  |  |  | **Sleep apnea symptoms** | | |  |  |
| --- | --- | --- | --- | --- | --- | --- | --- | --- | --- | --- | --- | --- | --- | --- | --- | --- | --- | --- | --- | --- | --- | --- | --- | --- | --- |
|  |  | ***β*** | | **(95 % CI)** | ***p*** | |  | ***β*** | | **(95 % CI)** | ***p*** | |  | ***β*** | | **(95 % CI)** | ***p*** | |  | ***β*** | | **(95 % CI)** | ***p*** | |  |
|  |  |  | |  |  | |  |  | |  |  | |  |  | |  |  | |  |  | |  |  | |  |
| AgeDev_Pheno_ |  | 0.21 | | -0.10, 0.51 | 0.187 | |  | 0.04 | | -0.33, 0.41 | 0.839 | |  | -0.25 | | -0.64, 0.14 | 0.203 | |  | 0.32 | | 0.00, 0.64 | 0.053 | |  |
| AgeDev_Grim_ |  | 0.09 | | -0.08, 0.26 | 0.320 | |  | 0.04 | | -0.16, 0.24 | 0.690 | |  | -0.11 | | -0.33, 0.10 | 0.304 | |  | 0.52 | | 0.34, 0.69 | **1.31e-8*** | |  |
| AgeDev_Hannum_ |  | 0.04 | | -0.20, 0.28 | 0.759 | |  | 0.01 | | -0.27, 0.30 | 0.935 | |  | -0.05 | | -0.35, 0.26 | 0.774 | |  | -0.05 | | -0.31, 0.20 | 0.692 | |  |
| AgeDev_Horvath_ |  | 0.07 | | -0.17, 0.30 | 0.595 | |  | 0.16 | | -0.12, 0.45 | 0.275 | |  | 0.13 | | -0.17, 0.44 | 0.394 | |  | 0.21 | | -0.04, 0.46 | 0.102 | |  |
| DunedinPACE |  | 0.01 | | 0.00, 0.01 | **0.017*** | |  | 0.01 | | 0.00, 0.01 | 0.143 | |  | -0.01 | | -0.01, 0.00 | 0.142 | |  | 0.02 | | 0.02, 0.03 | **5.44e-14*** | |  |
| Statistically significant associations (p < 0.05, unadjusted) are bolded, and those that remained significant after FDR correction are marked with an asterisk. The models were adjusted for array type, sex and smoking status. | | | | | | | | | | | | | | | | | | | | | | | | | |

**Supplementary Table 6.**

*Results of regression analyses when predicting epigenetic ageing measures by years spent in shift work.*

|  |  |  | **Model 1**  **(n = 667)** |  |  |  | **Model 2**  **(n = 616)** |  |  |  | **Model 3**  **(n = 658)** |  |  |
| --- | --- | --- | --- | --- | --- | --- | --- | --- | --- | --- | --- | --- | --- |
|  |  | ***β*** | **95 % CI** | ***p*** |  | ***β*** | **95 % CI** | ***p*** |  | ***β*** | **95 % CI** | ***p*** |  |
| AgeDev_Pheno_ |  | 0.02 | -0.04, 0.07 | 0.620 |  | 0.01 | -0.05, 0.07 | 0.638 |  | 0.02 | -0.05, 0.07 | 0.633 |  |
| AgeDev_Grim_ |  | 0.03 | 0.00, 0.06 | 0.063 |  | 0.02 | -0.01, 0.05 | 0.249 |  | 0.02 | -0.01, 0.06 | 0.167 |  |
| AgeDev_Hannum_ |  | -0.02 | -0.06, 0.03 | 0.421 |  | -0.03 | -0.08, 0.02 | 0.230 |  | -0.01 | -0.06, 0.03 | 0.580 |  |
| AgeDev_Horvath_ |  | 0.05 | 0.00, 0.09 | 0.044 |  | 0.04 | -0.01, 0.09 | 0.092 |  | 0.05 | 0.00, 0.10 | 0.051 |  |
| DunedinPACE |  | 1.3e-3 | 2.6e-4, 2.4e-3 | **0.016*** |  | 1.1e-3 | 7.60e-5, 2.1e-3 | **0.035** |  | 9.4e-4 | -1.8e-4, 2.1e-3 | 0.098 |  |
| Statistically significant associations (p < 0.05, unadjusted) are bolded, and those that remained significant after FDR correction are marked with an asterisk. Models 1 were adjusted for sex, daily smoking status and array type. Models 2 were adjusted for Model 1 covariates and health factors. Models 3 were adjusted with Model 1 covariates and socioeconomic factors. | | | | | | | | | | | | |  |

**Supplementary Table 7.**

*Results of regression analyses when predicting epigenetic ageing measures by hours of sleep.*

|  |  |  | **Model 1**  **(n = 1618)** |  |  |  | **Model 2**  **(n = 1461)** |  |  |  | **Model 3**  **(n = 1441)** |  |  |
| --- | --- | --- | --- | --- | --- | --- | --- | --- | --- | --- | --- | --- | --- |
|  |  | ***β*** | **95 % CI** | ***p*** |  | ***β*** | **95 % CI** | ***p*** |  | ***β*** | **95 % CI** | ***p*** |  |
| AgeDev_Pheno_ |  | -0.12 | -0.46, 0.22 | 0.479 |  | -0.06 | -0.41, 0.29 | 0.744 |  | -0.1 | -0.48, 0.27 | 0.592 |  |
| AgeDev_Grim_ |  | -0.19 | -0.38, 0.01 | **0.042** |  | -0.16 | -0.35, 0.03 | 0.091 |  | -0.17 | -0.38, 0.03 | 0.0966 |  |
| AgeDev_Hannum_ |  | -0.02 | -0.29, 0.24 | 0.857 |  | 0.04 | -0.24, 0.32 | 0.778 |  | 0.16 | -0.13, 0.46 | 0.281 |  |
| AgeDev_Horvath_ |  | -0.03 | -0.30, 0.23 | 0.808 |  | -0.09 | -0.38, 0.19 | 0.519 |  | 0.15 | -0.14, 0.45 | 0.311 |  |
| DunedinPACE |  | -0.01 | -0.02, 0.01 | **4.47e-5*** |  | -0.01 | -0.02, -4.2e-3 | **0.001*** |  | -0.01 | -0.02, -0.01 | **4.5e-4*** |  |
| Note: Models 1 were adjusted for sex, daily smoking status and array type. Models 2 were adjusted for Model 1 covariates and health factors. Models 3 were adjusted with Model 1 covariates and socioeconomic factors. | | | | | | | | | | | | |  |

**Supplementary Figure 1.**

*Pearson correlations between different indicators of epigenetic ageing and sleep measures.*

| 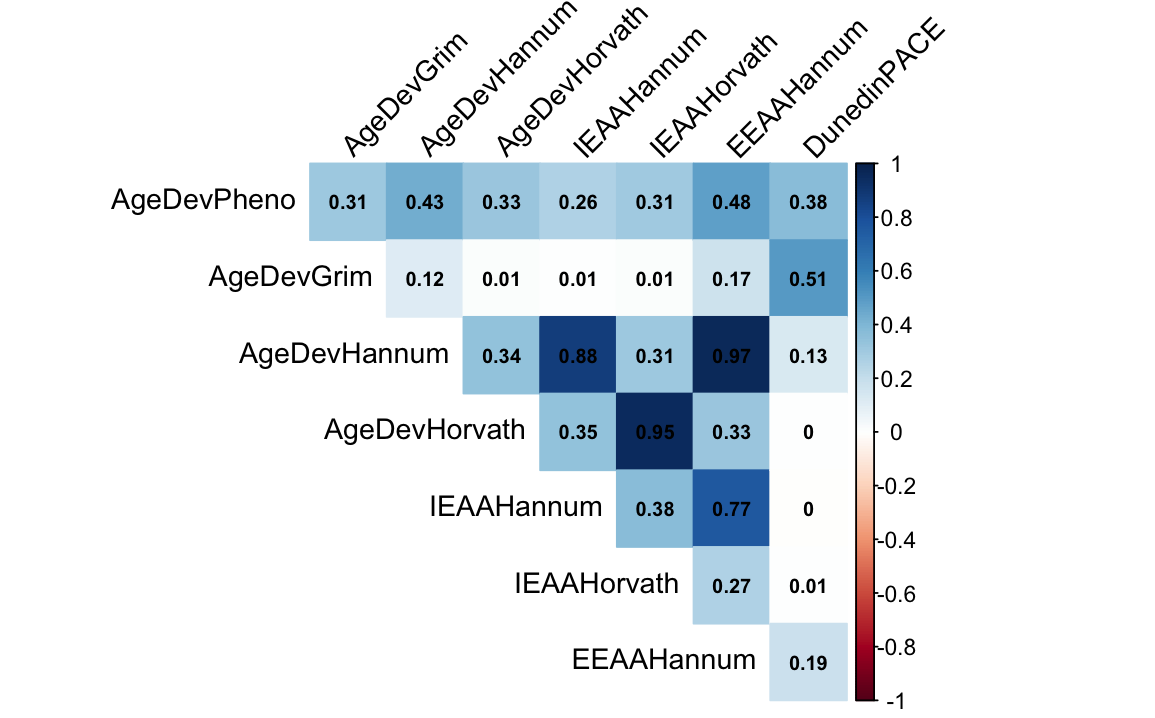 | 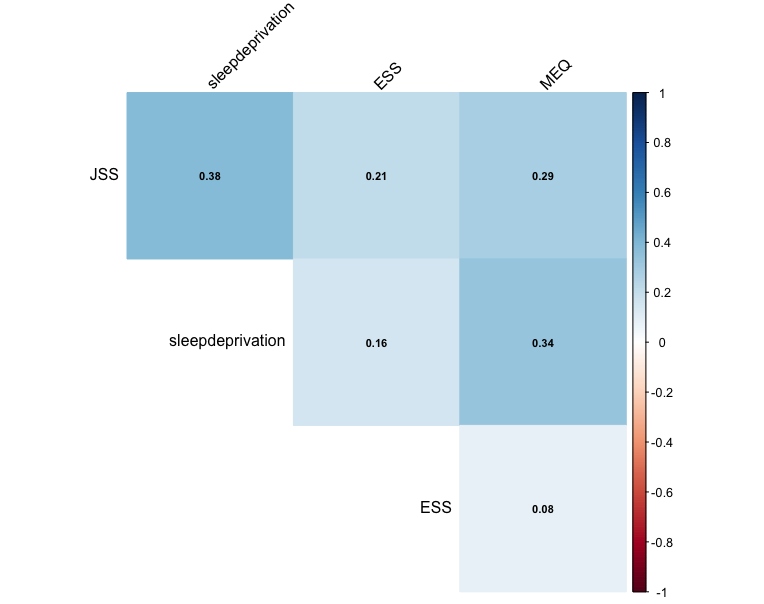 |
| --- | --- |

*JSS = the Jenkins Sleep Scale, ESS = the Epworth Sleepiness Scale, MEQ = the Morningness-Eveningness Questionnaire.*
